# Spiking Neural Network Modelling Approach Reveals How Mindfulness Training Rewires the Brain

**DOI:** 10.1038/s41598-019-42863-x

**Published:** 2019-04-23

**Authors:** Zohreh Doborjeh, Maryam Doborjeh, Tamasin Taylor, Nikola Kasabov, Grace Y. Wang, Richard Siegert, Alex Sumich

**Affiliations:** 1 Knowledge Engineering and Discovery Research Institute (KEDRI), Auckland University of Technology, Auckland, New Zealand; 2 Department of Psychology, Auckland University of Technology, Auckland, New Zealand; 3 Division of Psychology, Nottingham Trent University, Nottingham, United Kingdom

**Keywords:** Learning algorithms, Network models, Human behaviour

## Abstract

There has been substantial interest in Mindfulness Training (MT) to understand how it can benefit healthy individuals as well as people with a broad range of health conditions. Research has begun to delineate associated changes in brain function. However, whether measures of brain function can be used to identify individuals who are more likely to respond to MT remains unclear. The present study applies a recently developed brain-inspired Spiking Neural Network (SNN) model to electroencephalography (EEG) data to provide novel insight into: i) brain function in depression; ii) the effect of MT on depressed and non-depressed individuals; and iii) neurobiological characteristics of depressed individuals who respond to mindfulness. Resting state EEG was recorded from before and after a 6 week MT programme in 18 participants. Based on self-report, 3 groups were formed: non-depressed (ND), depressed before but not after MT (responsive, D^+^) and depressed both before and after MT (unresponsive, D^−^). The proposed SNN, which utilises a standard brain-template, was used to model EEG data and assess connectivity, as indicated by activation levels across scalp regions (frontal, frontocentral, temporal, centroparietal and occipitoparietal), at baseline and follow-up. Results suggest an increase in activation following MT that was site-specific as a function of the group. Greater initial activation levels were seen in ND compared to depressed groups, and this difference was maintained at frontal and occipitoparietal regions following MT. At baseline, D^+^ had great activation than D^−^. Following MT, frontocentral and temporal activation reached ND levels in D^+^ but remained low in D^−^. Findings support the SNN approach in distinguishing brain states associated with depression and responsiveness to MT. The results also demonstrated that the SNN approach can be used to predict the effect of mindfulness on an individual basis before it is even applied.

## Introduction

A substantial literature, including systematic reviews, supports mindfulness-based practices in the management of emotions and improved cognition^1–4^. Mindfulness training has thus been adopted by many sectors across a wide range of contexts including education, workplace, health care practices, and rehabilitation centres^5,6^. Self-report measures of state and trait mindfulness are commonly used as outcome measures. However, there has been a growing need to better understand associated neural mechanisms given the potential health benefits^7,8^. It is widely accepted that the human brain is capable of reorganisation and the generation of functional connections to compensate for deficits caused by injury, disease and aging^9–11^ and advances in neuroimaging have contributed to understanding the underpinning mechanisms of neuroplasticity and skill learning^12,13^. Accordingly, neuroimaging offers an opportunity to investigate the neural reorganisation associated with mindfulness training.

A recent systematic review supported Mindfulness-Based Interventions (MBIs) in modulating several brain regions implicated in the pathophysiology of depression, (e.g., prefrontal cortex, basal ganglia, cingulate and parietal cortices) and cognitive processes, such as self-awareness, sustained attention, visual-spatial memory, working memory and emotion regulation^1,8^. Neuroimaging studies also suggest that consistent mindfulness practice results in increased thickness of various cortical regions linked with auditory, visual, and somatosensory processing functions^14–16^. Subdomains of mindfulness may rely on distinct brain networks. For example, reduced nodal strength in the left posterior cingulate gyrus, bilateral paracentral lobule, and middle cingulate gyrus following MT may reflect self-detachment^17^. Zhuang *et al*.^18^ found that Describing, Non-judging, and Non-reactivity facets of mindfulness were selectively associated with the cortical volume, thickness and surface area of multiple prefrontal regions and the inferior parietal lobule^18^. Changes in the hippocampal-cortisol association following a compassion and mindfulness-based meditation were dependent on changes in awareness of experience subscores^19^.

Electroencephalography (EEG) offers high temporal resolution and provides a useful tool in investigating neural oscillations and connectivity^20^. EEG studies have shown alterations in several frequency bands that may be dependent on the type of meditation practice: focused attention, open-monitoring, transcendental meditation, and loving-kindness^21^. In the last several decades, the power spectrum of EEG have been found to provide information on depressive state as well as recovery. Numerous studies have found significant differences in depressive patient EEG sub-bands compared to healthy control^22–25^. For example, synchronisation of alpha and beta band frequencies from right inferior frontal and primary sensory areas may reflect increases in controlled attention^7^, also associated with increased theta (anterior and posterior^3,21^).

Given the complexity of various interacting networks that underpin behaviour^26,27^, a challenge in understanding brain function, measured as spatio-temporal brain data (STBD), is the integration of both spatial and temporal components. Hence, a proper unifying computational model is required to effectively model the integrated spatio-temporal relationship in such multivariate data.

EEG is a widespread non-invasive type of STBD that predominantly reflects the signal from the gross change in extracellular electrical potential from pyramidal cells. Information is conveyed in the synchrony and frequency of neuronal firing^20^ with very high temporal resolution (milliseconds). Although spatial resolution of EEG data is limited, important temporal information is derived from the spatial topography underpinned by activation of distinct neuronal clusters^26,28^. Temporal features in EEG data manifest complex interactions between spatially distributed neural clusters that change dynamically in time^29^.

To model the spatio-temporal interaction, Spiking Neural Networks (SNNs)^30,31^ are considered as promising models that can learn from changes in temporal information over time while preserving the spatial relationships of the data variables. Incorporating space and time components of data in the SNN model enables to perform multidimensional learning from data that can be interpreted through meaningful 3D visualisation, pattern recognition, and classification. Hitherto, various SNN models have been developed to enhance the analysis and understanding of STBD (e.g. pattern recognition and functional pathway identification in EEG and fMRI data)^32,33^.

Employing SNN, we propose a method and a computational model that aims to investigate the effects of MT on both groups and individual subjects using EEG data. The method was applied to address the following objectives:

1. To recognise the patterns of changes in STBD, measured before and after MT across participants with different levels of depression.
2. To investigate whether STBD can identify if a person is likely to benefit from MT.

The population study contains three groups of participants who underwent MT, characterised by (a) non-depressed (denoted as ND); (b) high depression scores prior to training who showed a reduced level of depression scores post-MT (denoted as D^+^); and (c) high depression scores pre-MT who declared no changes in depression scores post-MT (denoted as D^−^).

For deeper analysis of the effects of MT on EEG data, rhythms of delta (δ), theta (θ), alpha (α) and beta (β) bands were extracted and separately analysed. These EEG frequency sub-bands were visualised by paying special attention to the underpinning brain activity changes among the D^+^ group. Finally, the SNN-based methodology was used for prediction of response to MT in individuals from depressed group, when only the EEG data from the pre-mindfulness stage was used.

## Method and Procedure

### Ethics

All experiments were performed in accordance with the relevant guidelines and regulations. Ethical approval was obtained by the Auckland University of Technology (AUT) Ethics Committee (AUTEC) New Zealand, and informed consent obtained.

### Participants

Forty participants underwent clinical assessment, including (BDI-II)^34^. To model changes in brain function as a result of the MT, specific participants were selected according to their scores on the depression subscales. A total of 18 participants (6 males with a mean age of 27.50 (SD = 7.34) years and 12 females with a mean age of 28 (SD = 10.43) years) were selected for the EEG data analysis.

In this dataset, we defined three classes: ND, D^+^ and D^−^, which were labelled according to the BDI-II test results reported in Supplementary Table 1. In class 1, seven participants who scored lower than 10 in BDI test were selected. In class 2, six participants affected by moderate level of depression who have responded to the training were selected. In class 3, five participants with the clinical and severe level of depression who have not responded to the training were selected. Two stages of EEG data recording were conducted in this research. The first stage was related to EEG data acquisition at baseline (denoted as T1) and the second one was recorded after a 6-week webinar mindfulness programme (denoted as T2) from all the participants.

Descriptive information of all participants including age, gender, mean age, range, mean score and the standard deviation along with descriptive information of BDI test, including mean and standard deviation are reported in Supplementary Table 2 and 3.

### Mindfulness Training

The mindfulness program was modified from an educational mindfulness program called Pause, Breathe and Smile^35^. Each session lasted 90–110 minutes and predominantly comprised a brief guided meditation exercise and a mindful tasting exercise. A detailed description of the program and effect on mood has been explained in previous research^36^.

### EEG Data Collection

EEG were recorded in two sessions (1) before participants began training in MT and (2) following 6 weeks of MT. In each session, resting state data were recorded for 2 minutes with eyes-closed.

Recordings were carried out using a SynAmps amplifier and 62-channel QuikCap with electrode placements based on standard 10–20 international system. The EEG channels are: FP1, FPZ, FP2, AF3, AF4, F7, F5, F3, F1, FZ, F2, F4, F6, F8, FT7, FC5, FC3, FC1, FCZ, FC2, FC4, FC6, FT8, T7, C5, C3, C1, CZ, C2, C4, C6, T8, TP7, CP5, CP3, CP1, CPZ, CP2, CP4, CP6, TP8, P7, P5, P3, P1, PZ, P2, P4, P6, P8, PO7, PO5, PO3, POZ, PO4, PO6, PO8, CB1, O1, OZ, O2, CB2. Data was recorded at a sampling rate of 1000 Hz. Off-line ICA computerised artefact correction was used to remove detectable eye movement or muscles potentials.

### EEG Data Pre-Processing

The EEG data were divided into two conditions (T1 and T2) across all 18 participants (11 depressed participants and 7 non-depressed participants). The EEG data were processed by down sampling to 500 Hz and using a 30 second sliding temporal data window, creating 60 samples per each of the two conditions.

To investigate the effects of mindfulness on the EEG frequency sub-bands, the EEG signals were divided into bands of type δ, θ, α and β by using a set of power-pass filtering in MATLAB through Fast Fourier Transform (FFT)^37^.

Supplementary Table 1 reports the participants’ information and their BDI scores from three groups: either D^+^ participants, or D^−^ and a ND group. For example, participant Id: S1 (from D^+^ group) scored 20, at T1, and 8, at T2 in the BDI- II test. This indicates a clinically significant improvement in the depression scores with changes from moderate level of depression to a normal healthy level. On the other hand, participant Id: S#2 (from D^−^ group) scored 36 at T1, and 22 at T2 in the BDI subscale. This indicates the participant scores remained at the depressed level despite it changing from severe to moderate.

### The Proposed Method and SNN Computational Architecture for Modelling and Comparative Analysis of Brain States Using EEG Data Collected Before and After Mindfulness Training

**Figure 1:**
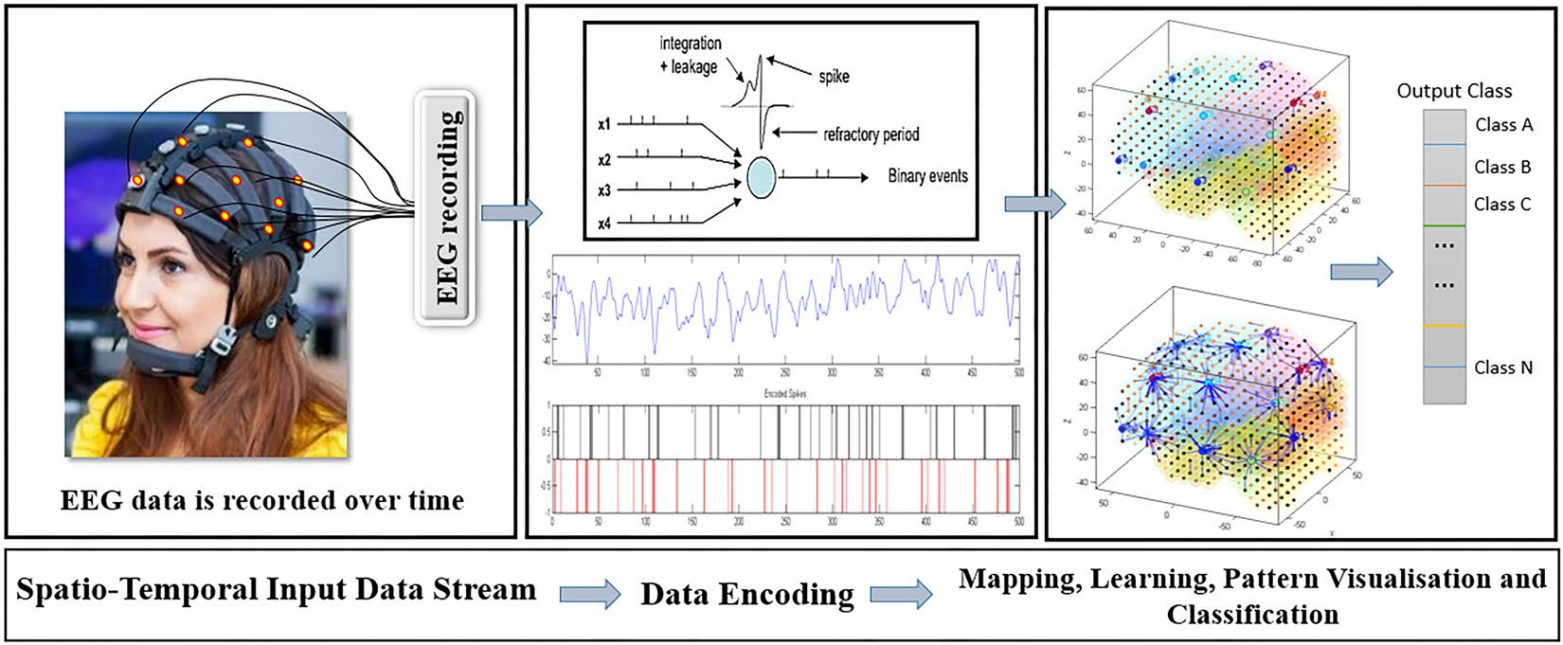
A block diagram of the proposed method, consisting of: encoding EEG data into spike sequences; a brain-inspired 3D SNN structure for data mapping; learning; visualisation in 3D SNN; and output classification of patterns. (NB. Model used in photograph). (The person in this figure is the original picture of M. Doborjeh and taken by Z. Doborjeh who are the authors of this paper).

The SNN method and its computational architecture proposed in this study include several algorithms that allow for aspects of EEG data to be comprehensively evaluated. The architecture consists of the following main modules, each of them includes several algorithms for building a particular model of STBD (shown graphically in Fig. 1):

- **Data encoding:** Spatio-temporal EEG data were measured as temporal sequences of continues real values, which in our study were converted (encoded) into discrete spikes. In the example shown in Fig. 1, we used a threshold-based method^38^ for EEG encoding. In this method, if the signal’s change increases above a spike threshold at consecutive time moment, a positive spike is generated. On the other hand, if the signal decreases bellow a threshold, then a negative spike is generated; otherwise, there is no spike generated.
- **Mapping:** For the mapping of EEG data into a 3D SNN reservoir, the Talairach brain template^39,40^ was used. The input EEG data variables were positioned in the SNN model as input neurons with respect to their ($x,{y},{z}$) coordinates as located in the Talairach brain atlas.
- **Learning:** After an SNN model was spatially mapped, the model was trained in an unsupervised learning mode by using a spike-timing learning rule (in this case, using the Spike Time Dependent Plasticity (STDP) learning rule)^41^. In this study, different SNN models were trained with the EEG data related to different mental states, e.g. before (Time T1) and after (Time T2) MT, across groups of participants. The SNN models of T1 and T2 were subtracted to capture the differences between the two states as a result of MT.
- **Pattern visualisation:** To better understand the modification of spatio-temporal interactions between brain areas (62 EEG channels) in relation to MT, the SNN models were visualised into a 3D space. We visualised six SNN models, each of them was trained separately by the EEG data of one group (ND, D^+^ and D^−^) at T1 and T2. The quantitative information of the visualised models were statistically analysed to investigate the effects of mindfulness on EEG data.
- **Pattern classification:** To perform a classification task, an output layer classifier was trained, at a supervised mode, to learn the association between the trained SNN connectivity and the class label information. This is performed here using the dynamic evolving Spiking Neural Network (deSNN) classifier^42^. This procedure was performed here for classification of EEG data (frequency sub-bands) into two classes T1 and T2 to investigate how the EEG were changed after the MT.
- **Prediction:** In order to predict whether an individual is likely to respond to MT at T2, we performed a classification task on the EEG data (from T1) into two classes D^+^ and D^−^ (assessed at T2).

### Compliance with Ethical Standards

This manuscript is the authors’ original work and has not been submitted for publication elsewhere and all experiments were performed in accordance with relevant guidelines and regulations.

## Results

The current study was organised in a two-phase analysis as follows:

1. EEG data were modelled using the SNN architecture above to investigate the effects of MT across participants with different mental health conditions.
2. Statistical analysis of the results to evaluate the model significance.

These steps are explained in the following sections 3.1 and 3.2.

### EEG Data Modelling using the proposed SNN Architecture

**Figure 2:**
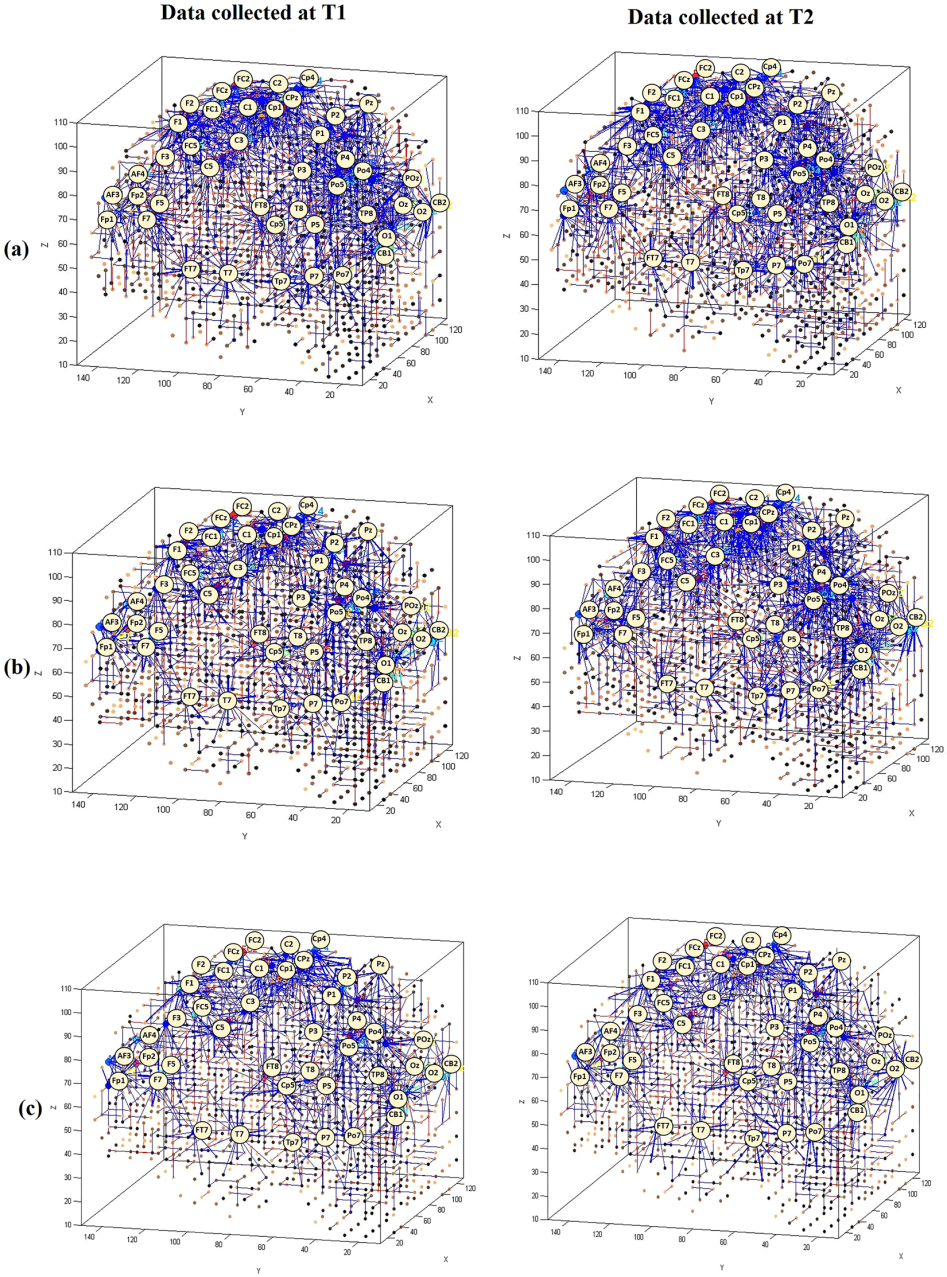
SNN connectivity trained on EEG samples that measured at T1 (before MT) and T2 (after MT), related to (**a**) non-depressed group (ND), (**b**) responsive-depressed group (D^+^) and (**c**) unresponsive-depressed group (D^−^).

The 62 EEG channels were mapped into a 3D space of 1471 artificial spiking neurons, where the spatial locations of the input neurons were the same as their (x, y, z) coordinates in the Talairach brain template^39,40^. Figure 2 illustrates the SNN models were trained on EEG data related to before and after MT (T1 and T2) across three groups of participants. It shows differences in the development of spatio-temporal connections in the SNN models as a function of group (ND, D^+^ and D^−^).

To better scrutinise the differences between the SNN models of different mental states, the connection weights (*W*_*ij*_) of the difference between two correspondingly trained SNN models (T1, T2) was calculated for each group and subtracted (*W*_*ij*_*T*2 − *W*_*ij*_*T*1). The total connection weight of each SNN model in three groups are reported in Table 1.

**Table 1:** The average of the connection weights for each SNN model as an activation metric for three groups of participants: non-depressed (ND); responsive-depressed (D^+^); and unresponsive depressed (D^−^) over two time points: before training (T1) and after training (T2).

Average Connection weight of the SNN models		
Group	Time point	
	T1	T2
ND	0.84	1.04
D+	0.74	0.89
D−	0.54	0.64

**Figure 3:**
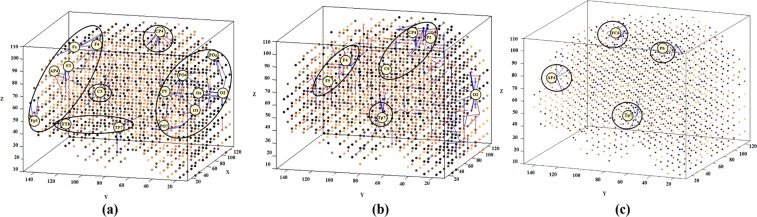
Differences between the connectivity in the trained SNN models of T1 (prior to MT) and T2 (post-training) in (**a**) non-depressed (ND) group, (**b**) responsive-depressed (D^+^) group, and (**c**) unresponsive-depressed (D^−^) group. The connections in each neural cluster represent the areas of main changes in the EEG data at post-MT.

**Figure 4:**
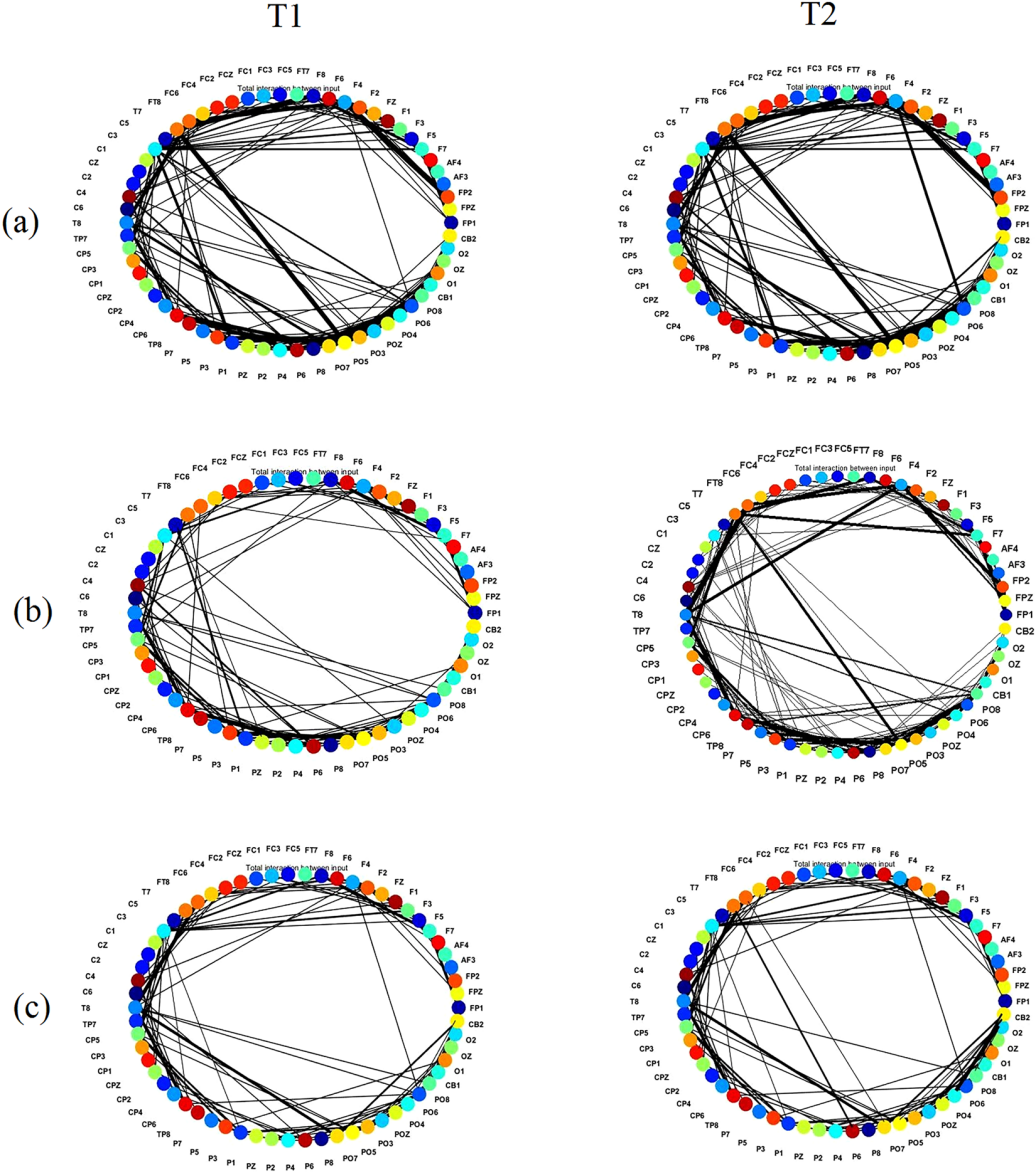
The Feature Interaction Network (FIN) captured the total spike interaction between the areas in the SNN models representing 62 EEG channels as input neurons during the STDP learning for: (**a**) non-depressed (ND); (**b**) responsive-depressed (D^+^); and (**c**) unresponsive depressed (D^−^). FIN nodes represent the input neuronal areas of the SNN model and lines represent the amount of spike transmission between these areas (clusters) of neurons that correspond to the input neurons (EEG channels).

The subtracted connectivity model is depicted in Fig. 3 which shows the involved brain areas, activated in response to the MT. The differences between the SNN models of T1 and T2 can be also studied by computing the amount of spatio-temporal interactions between the EEG variables using a Feature Interaction Network (FIN). In Fig. 4, the total temporal spike interaction among 62 input neuronal areas (corresponding to 62 EEG channels) is shown in the FIN, where nodes represent the input neuronal areas (neuronal clusters) and each line, that links two nodes, corresponds to the amount of spike transmission between the clusters during the SNN learning model.

#### Analysis of Functional Changes across Power Band-Frequency

**Figure 5:**
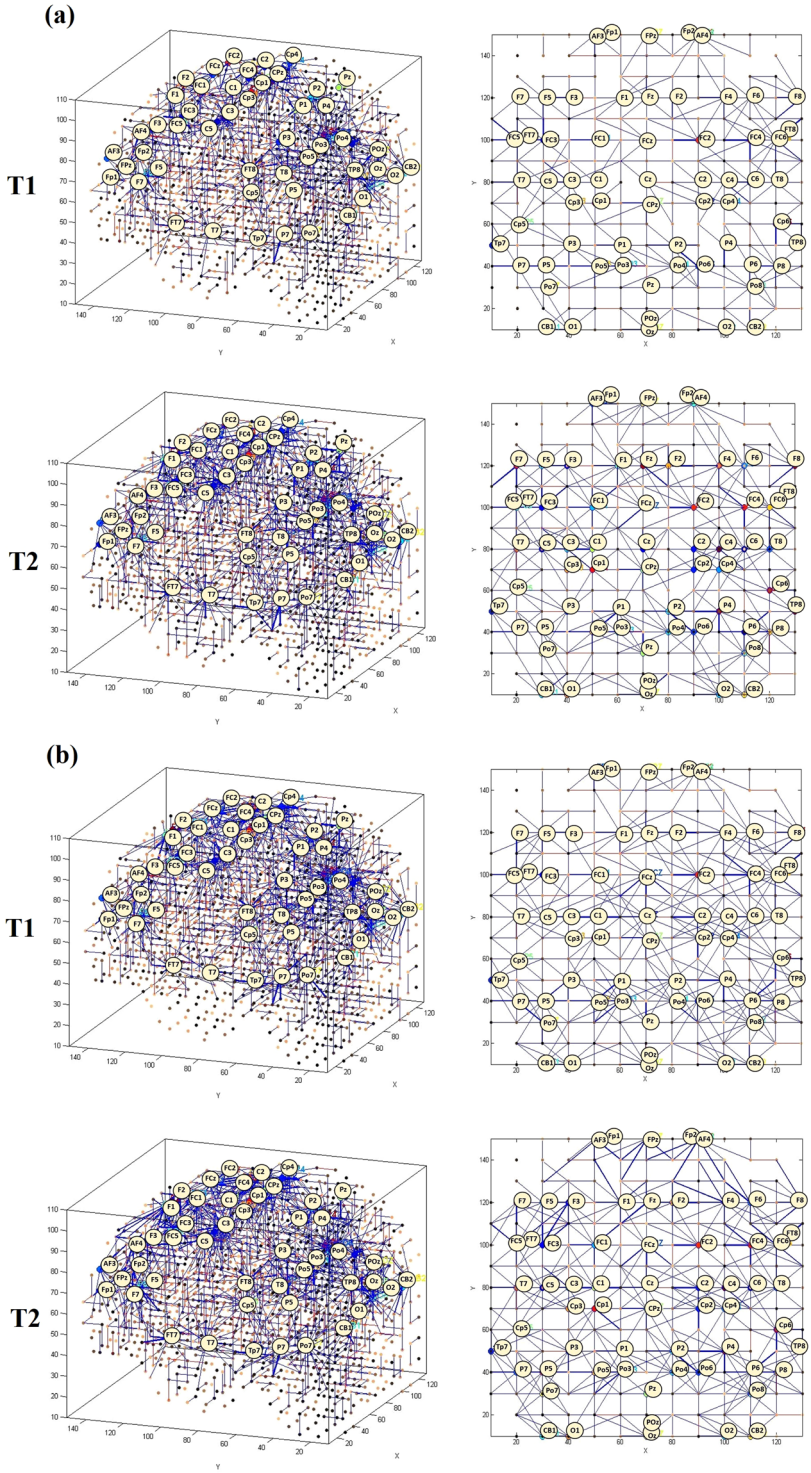
Spatio-temporal connectivity generated in the SNN models for responsive-depressed (D+) participants. The SNN models are visualised in both 3D (x, y, z) and 2-D (x, y) projections. (**a**) Delta frequency sub-band at T1 and delta at T2 (**b**) theta at T1 and theta at T2.

**Figure 6:**
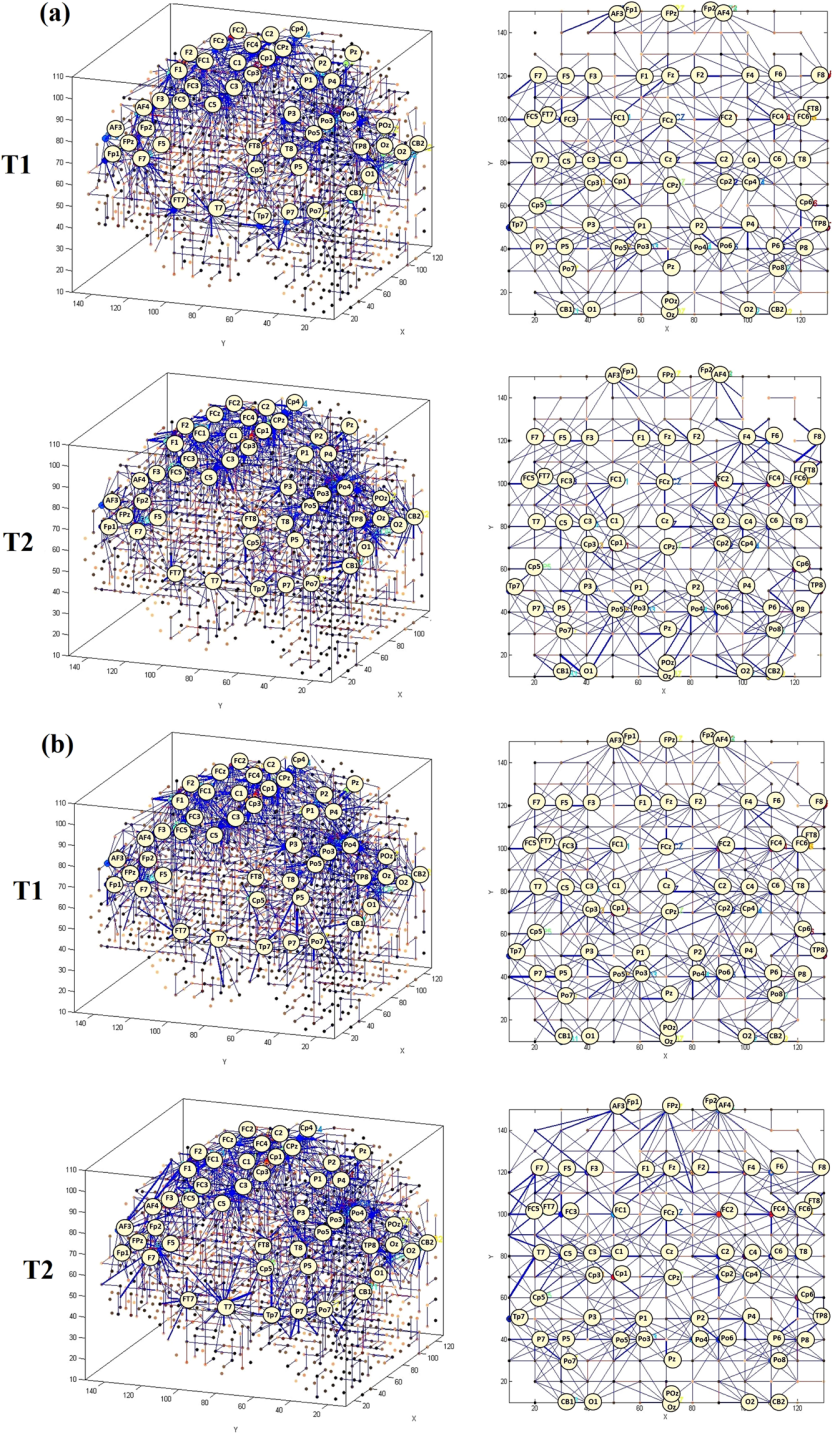
Spatio-temporal connectivity generated in the SNN models for responsive-depressed (D+) participants. The SNN models are visualised in both 3D (x, y, z) and 2-D (x, y) projections. (**a**) Alpha at T1 and alpha at T2, (**b**) beta at T1 and beta at T2.

**Figure 7:**
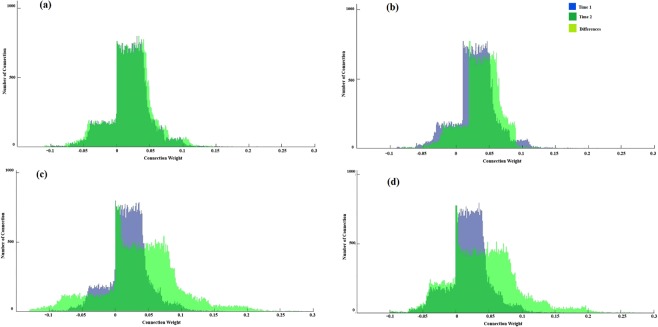
Histogram of the number of connections and the connection weights in the SNN models for the D^+^ group trained on data corresponding to four EEG frequency sub-bands before (T1) and after (T2) MT. (**a**) Delta, (**b**) Theta, (**c**) Alpha, and (**d**) Beta.

For a deeper examination of the effect of MT on the participants in the depressed group, we developed the SNN models of different EEG frequency sub-bands, including delta (δ: 0.4–4 Hz), theta (θ: 4–8 Hz), alpha (α: 8–12 Hz), and beta (β: 12–28 Hz), shown in Fig. 5 and Fig. 6. The visualisation of EEG sub-bands as a function of time (T1, T2) in depressed participants-group, suggested that alpha and beta bands were most affected by the MT based. Table 2 reports the connection weight of four sub-bands SNN models. Further quantitative information of the differences between the SNN models of these four EEG frequency sub-bands are plotted in Fig. 7. This illustrates the SNN models’ histograms, defined by the number of connections as well as the connection weight in the SNN models of different sub-bands related to before and after the MT.

**Table 2:** The average of the connection weights for each SNN model of sub-bands frequency for the responsive-depressed (D^+^) group before training (T1) and after training (T2).

Average Connection weight of the sub-bands SNN models			
Group	bands	Time point	
		T1	T2
D+	Delta	0.7	0.7
	Theta	0.79	0.98
	Alpha	1.02	1.26
	Beta	1.03	1.17

#### Pattern Classification of EEG Data Measuring Brain States Before and After Mindfulness Training in the Designed SNN Model

Here, we trained an output classifier to investigate how well a brain-inspired SNN model can classify the EEG patterns of T1 and T2 stages. There was a total number of 120 EEG samples (60 samples per class) for the classification task, which was here based on a leave-one-out cross-validation (LOOCV) method. The classification experiment was performed four times, separately for each EEG frequency sub-bands as reported in Table 3. An essential step in finding optimal results from the SNN model is the optimisation of its parameters. Therefore, SNN training and validation procedures were repeated in a LOOCV mode with different combinations of parameter values in a grid-search, with an objective function being a highest classification accuracy. Table 3 indicates the total classification accuracy of EEG frequency sub-bands as a function of time (T1, T2).

**Table 3:** Classification accuracy of 120 EEG samples (10 samples per participant) from six D^+^ participants at T1 (class 1) and T2 (class 2), performed using four EEG frequency sub-bands (modelled separately). The diagonals on the confusion tables represent the correctly classified samples. Highest classification accuracy is seen for alpha (91%) and beta (85%).

EEG frequency sub-bands			Accuracy	F-score
Delta				
EEG Data Classes	Delta (T1)	Delta (T2)	0.75	75%
Delta (T1)	**36**	24		
Delta (T2)	5	**55**		
**Theta**			**Accuracy**	
EEG Data Classes	Theta (T1)	Theta (T2)	0.81	81%
Delta (T1)	**42**	18		
Delta (T2)	4	**56**		
**Alpha**			**Accuracy**	
EEG Data Classes	Alpha (T1)	Alpha (T2)	0.91	90%
Delta (T1)	**51**	11		
Delta (T2)	1	**59**		
**Beta**			**Accuracy**	
EEG Data Classes	Beta (T1)	Beta (T2)	0.85	85%
Delta (T1)	**44**	16		
Delta (T2)	1	**59**		

#### Prediction of Response to Mindfulness Training

To investigate whether the SNN architecture can be used for prediction of response to MT, we trained an SNN model using only the EEG data collected at T1 to predict the output classes at T2. The predictive outcomes were here the two groups of participants (D+and D−). For each participant, we extracted 10 samples in EEG data, each of them had a length of 1000 time points (one second EEG recording at resting state at T1). In total, the classification task was performed using 110 EEG samples, which belonged to six D^+^ participants as class 1 and five D^−^ participants as class 2. The classification was based on the (LOOCV) method and the results are reported in Table 4.

**Table 4:** Classification accuracy of 110 EEG samples from T1 (10 samples per participant) into two classes D^+^ and D−. There were six D^+^ participants as class 1 and five D^−^ participants as class 2. This is to predict which participant is likely to response to the MT at time T2 (after training) when the SNN model was only trained by the EEG data from T1. The diagonal on the confusion table represents the correctly predicted samples.

Predicted	D^+^ at T1	D^−^ at T1	Accuracy Per Class	Total Accuracy	F-score
Real					
D^+^ at T1	**54**	6	0.90	**0.87**	**89%**
D^−^ at T1	5	**42**	0.84		

### Statistical Analysis of the SNN Results

We calculated the average weight of the connections for each of the trained SNN models and reported this activation level as a function of group and time point (Table 1). As shown in Table 1, the SNN models of ND participants represented the strongest average activation levels (T1 = 0.84, T2 = 1.04), compared to D^+^ participants (T1 = 0.74, T2 = 0.89), and D^−^ participants (T1 = 0.54, T2 = 0.64).

We then clustered the EEG channels into five sites with respect to their topography (Supplementary Figure 1): frontal, frontocentral, temporal, centroparietal and occipitoparietal. Supplementary Table 4 reported the average weight of the connections that were formed in each site of the SNN models, again as a function of group and time point. Supplementary Figures 2,3 and 4 show the line graphs of average activation levels for each EEG channels as a function of *Group*, *Site* and *Time*.

**Figure 8:**
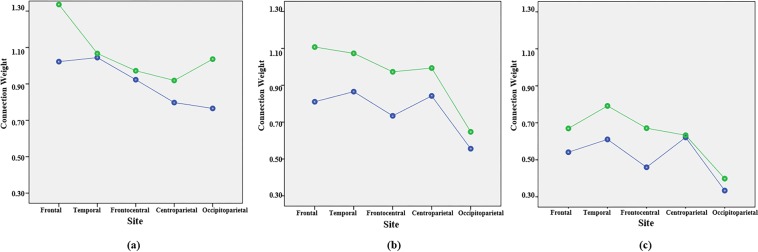
The SNN connection weights prior to MT (T1) and after following 6 weeks of training (T2) in (**a**) ND group, (**b**) D^+^ group and (**c**) D^−^ group at Frontal, Temporal, Frontocentral, Centroparietal and Occipitoparietal clusters. Blue line represents the connectivity values in the SNN model of T1 (before mindfulness training) and green line represents T2 (after the mindfulness training).

For each participant from the three groups (ND, D^+^, D^−^), we developed one SNN model and trained it with the EEG data of this participant. Repeated-measures Analysis of Variance (ANOVA) tested for differences in activation levels as a function of three within-subjects variables, including, *Hemisphere* (left, right), *Time* (T1, T2) and *Site* (frontal, frontocentral, temporal, centroparietal and occipitoparietal), and *Group* (ND, D^+^, D^−^). Figure 8 shows the line graphs of average activation levels as a function of *Group*, *Site* and *Time*. Supplementary Tables 5 and 6 show descriptive (means, SDs) as a function of *Group*, *Site*, *Hemisphere* and *Time*.

Results of the repeated-measures ANOVA are presented in Table 5, which shows a significant main effect of *Time* (T1 < T2) and *Group* (ND > D^+^ > D^−^). The significant Site*Group interaction suggested that ND participants had higher activation values than D^+^ at frontal and occipitoparietal sites. Higher activation values were seen for ND and D^+^ compared to D^−^ at all sites. The significant Site*Time*Group interaction appeared to be due to an increase in activation levels at T2 compared to T1 across frontal, centroparieal and occipitoparietal for the ND group. For D^+^ group, the effect of *Time* (T2 > T1) was seen at all sites except occipitoparietal. For D^−^ group, the effect of *Time* (T2 > T1) was seen at all sites except centroparietal and occipitoparietal. Also, there was a Site*Group interaction at T2, but not at T1. This was because the effect of *Group* (ND > D^+^ > D^−^) at T1 was present at all sites. However, at T2 ND > D+ was only seen at frontal and Occipitoparietal sites; and at temporal sites no difference was seen between ND and D^+^.

**Table 5:** Repeated-measures ANOVA.

	Lower order Conditions	F-value	Degree of freedom	p-value	Eta^2^	Post Hoc Tests
Time		176.90	1,15	<0.001	0.92	T2 > T1
Group		44.81	2, 15	<0.001	0.86	ND > D + > D-
Site*Group		2.64	5.86, 43.93	0.029	0.26	(F): ND > D + > D- (FC): ND = D + > D- (T): ND = D + > D- (CP): ND<D + > D- (OP): ND > D + > D-
Site*Time*Group		2.89	6.52, 48.90	0.015	0.28	
Site*Time	ND	6.88	2.67, 16.02	0.004	0.53	Effect of Time (T2 > T1) (F): [F(1,6) = 82.90, p < 0.001, eta^2^ = 0.93] (CP): [F(1,6) = 11.72, p.014, eta^2^ = 0.66] (OP): [F(1,6) = 21.50, p = 0.004, eta^2^ = 0.78] Other sites: Not Significant
	D+	1.45	2.65, 13.24	0.273	0.22	T2 > T1 at all sites, except (OP)
	D−	1.36	1.66, 6.63	0.312	0.25	T2 > T1 at all sites, except (CP)and (OP)
Site*Group	T1	1.57	5.35, 40.12	0.153	0.17	ND > D + > D- at all sites
	T2	3.57	5.84, 43.78	0.006	0.32	(F): ND > D + > D- (FC): ND = D + > D- (T): ND = D + = D- (CP): D + > ND > D- (OP): ND > D + > D-
Time*Group	F	2.03	2,15	0.165	0.21	
	FC	3.41	2,15	0.060	0.31	
	T	2.90	2,15	0.086	0.28	
	CP	3.27	2,15	0.066	0.30	
	OP	3.62	2,15	0.052	0.32	Effect of Time (T2 > T1) ND[F(1,6) = 21.51, p = 0.004, eta^2^ = 0.78] D- [F(1,4) = 28.24, p = 0.006, eta^2^ = 0.88] D+ Not Significant

*p < 0.05

Frontal (F), Frontocentral (FC), Temporal (T), Centroparietal (CP) and Occipitoparietal (OP)

Not depressed group (ND), participants in depressed group who responded to the training (D^+^), participants in depressed group who did not respond to the training (D^−^).

Before training (T1) and after training (T2).

## Discussion

The present study proposed a brain-inspired SNN architecture for investigating neural activity as a function of depression and response to MT in a nonclinical population. The SNN models are used to determine the discriminative patterns in the EEG samples that recorded prior to and following a 6 week MT programme in three participant groups.

The proposed SNN architecture is performed through an empirical study that involved the following steps:

- Mapping the spatial information of EEG variables (channels), used to measure the effect of MT, to a 3D brain-inspired SNN model, pre-structured with the use of a standard brain template;
- Unsupervised learning of the spatially mapped EEG data in the SNN model using spike time dependent plasticity learning algorithm;
- Visualising and interpreting of the trained spatio-temporal connectivity of the SNN model;
- Classification, prediction and validation of the model;
- Statistical analysis of the models to evaluate the level of significance.

The research outcomes took into account the following sections 4.1 and 4.2:

### SNN Architecture for Modelling of Brain Mental States Followed by Mindfulness Training

To begin with, six SNN models were separately trained using different EEG data sample sets corresponding to: ND group at T1 and T2, D^+^ group at T1 and T2, and D^−^ group at T1 and T2. Figure 2 illustrates that the SNN connections have been evolved differently during the unsupervised learning with EEG data related to different brain mental states. The SNN models trained in each group revealed information about the involvement of particular brain areas after the MT.

To perform a better analysis of EEG changes after MT, we computed the differences between the SNN models of pre-MT and post-MT for each group through subtracting the two correspondingly trained SNN models (T1, T2). This allows to visualise the changes in neural connectivity as a result of MT over time. As shown in Fig. 3, our findings suggested that mindfulness resulted in a similar pattern of changes in some regional activation (frontal, frontocentral, and temporal) across all the three groups. However, the size of the activated connectivity was higher in the ND group, compared to D^+^ and D^−^, and that of D^+^ was higher than D^−^.

A within-group examination suggests that in ND group, MT was found to increase the spatio-temporal connectivity over the frontal (AF4, Fp1, F3, F4, Fz,), centroparietal (C5, CP4) and occipitoparietal areas (POz, PO7, PO4, O1, O2, Oz) at T2. For the D^+^ group, stronger connectivity at T2 was seen over posterior temporal areas (Tp7). However, the intensity of this connectivity was minor when compared with the ND group. Greater activation appeared over centroparietal regions (C6, CP4, and P2), whilst very little change was seen posteriorly (O2). For the D^−^ group, slight changes in SNN connectivity over frontal (AF4, F6), frontocentral (FC6) and temporoparietal (TP6) regions were observed. In total, the SNN model of D^−^ group has shown minimal changes after the MT. The fact that these participants did not perceive and/or did not report that the training had any effect on them and yet there were clear brain connectivity changes according to our modelling, may indicate that neural changes are not resulting a subjective change in depression^43,44^. That is, it has been theorised that core processes of implicit reactions may occur independently of conscious awareness^44^, but are detectable using brain imaging. SNN appears to be useful in this respect.

In order to analyse the information interaction between the brain areas in response to MT across the three groups, the total temporal interactions (in terms of spike communication) between 62 input neurons was depicted. Figure 4 shows the average one-to-one interaction between the inputs neurons (EEG cannels). As illustrated by the FIN graph in Fig. 4(a), broader interaction lines were formed between the 62 EEG channels of ND participants when compared with the D^+^ and D^−^ groups in Fig. 4(b,c). In FIN graph of D^+^ group, there were thicker interaction lines at T2, especially between the EEG channels positioned at frontal areas (Fp1, Fp2, AF3, AF4 and F7), when compared with the graph at T1 (before training). Thicker lines indicate more interaction between the inputs. These connections were established strongly because of more spikes transmitted between the neurons located in these areas, reflecting more changes in the corresponding EEG signals. In Fig. 4(c), it was revealed that although D^−^ participants reported that they did not respond to the MT (based on BDI-II scores), the FIN of the corresponding SNN model at T2 (after training) showed stronger interactions than T1 in brain areas in frontal, frontocentral and temporal.

In the current study, to precisely evaluate that how MT influenced the level of depression in D + group, we examined the EEG frequency sub-bands. As can be seen from Fig. 6(a,b), the SNN models of alpha and beta bands were more affected by the MT at T2 when compared with other frequency bands (Fig. 5). The SNN connectivity of alpha and beta bands at T2 show that stronger activity was observed at T2 (activation level = 1.26 and 1.17) than T1 (activation level = 1.02 and 1.03) in comparison with other bands. There was a significant difference at the electrode channels of F8, F4, O1, O2 and OZ in the SNN connectivity of alpha brainwaves at T2 as compared to the T1. These electrode channels located at the right frontal and occipital lobes. These areas involve in the motor planning, emotional expression, visual and sensory input processing from the environment. Lower SNN connectivity of alpha at T1 than T2 in these areas could be due to the less attentiveness to the outer environment. This finding is supported by previous empirical studies^45–47^ that suggested depressed people tend to more focus inward on own negative emotions. Therefore, they pay less attention on receiving the information from the outer world. The SNN model of beta band at T2, illustrate stronger connectivity around channels of F5, F7, AF3, Fp1 and T7 than other areas. These channels located at the left frontal and left temporal regions that are involved in verbal and emotional expression, attention and emotional memory^48^.

The SNN model of theta sub-band at T2 (Fig. 5b) shows that the spatio-temporal connectivity slightly increased over F3 and C3 channels that are located in left frontal and left central as compared with T1. These areas are engaged with motor planning and sensorimotor integration.

For the delta sub-band (Fig. 5a), all the connections in the SNN models of T1 and T2 were approximately uniform. Therefore, we could not distinguish specific modified connections in any parts of the SNN model of T2 that could suggest a response to the MT.

Our results are in line with the findings in literature that showed regional hemispheric asymmetries are associated with less activity in left frontal and right posterior regions in depression^49^^,^^50^^,^^51^. Decreased connectivity of the frontal regions has been suggested to account for loss of interest, motivation, and pleasure. These symptoms are typical characteristics in depression. Interactions among regions in this network have been shown to be attenuated in patients with depression, that all can be control of negative thoughts and emotions in depressed individuals^52,53^.

In our research, after MT, SNN connectivity of the frontal regions significantly increased. Stronger functional connectivity indicate an increased interplay of activated brain areas underlying cognitive functions^54^.

To validate the visualised changes in the SNN models that depict the variation in EEG sub-bands from T1 to T2, we performed a quantitative analysis on the SNN connection weights. We plotted eight histograms of the connection weights in the SNN models, each of which was separately trained by the EEG frequency sub-bands from T1 and T2 of the D^+^ group. Figure 7(a) represents that the SNN connection weight histograms related to the delta sub-band at T1 and T2 were overlapped. This supports our previous argument (shown in Fig. 5a and Table 2) that no significant changes were observed after the MT in the delta sub-band. However, the connection weight histograms of the other sub-bands varied to different degree from T1 to T2 as shown in Fig. 7(b–d).

We also performed a classification task on the EEG data sub-bands to examine if the SNN model can classify the EEG patterns of T1 and T2 stages. Table 3 shows that applying SNN for classification of spatio-temporal data resulted in significantly high accuracy for both alpha and beta sub-bands. This indicates that alpha and beta were the most affected EEG sub-bands after MT; therefore, the correspondingly trained SNN models could accurately classify between T1 and T2.

The SNN architecture was also applied in this research for prediction of response to MT. At this point, the SNN models were only trained by the EEG data from T1 (prior to training) to detect, in an earlier stage, whether a participant is likely to respond to the MT at T2. This was performed by classifying the EEG data of T1 into two classes of participants (D^+^ and D^−^ which were assessed at T2). Table 4 shows that the classification accuracy of EEG samples to D^+^ and D^−^ groups was 87%, which suggested the existence of discriminative patterns between the groups at T1. This finding indicates that the designed SNN-based methodology can be potentially explored and used in the future to predict response to treatment for depressed individuals before training is applied.

### Statistical Analysis of the SNN Results

For statistical analysis, the average value of connection weights in each trained SNN model were calculated and reported as an activation level towards each mental state (prior to and after MT). The ANOVA statistical analysis is then applied to represent the significance of the models. As reported in Table 1, we obtained a higher activation level of 1.04 in the trained SNN model that corresponds to ND group at post-training evaluation. Further information regarding this analysis can be obtained from the Supplementary Table 4 in which the average connection weights for every single site are reported for factors of *hemisphere* (left, right) and *Time* (before, after training) across all *groups* of participants. It shows higher average of connection weights over the right hemisphere at T2 for all the three groups of participants (1.15, 0.95 and 0.62) when compared to the right hemisphere at T1 (0.95, 0.76 and 0.51).

ANOVA analysis of the SNN models identified common changes under the MT across participants, but also those that varied as a function of group and responsiveness to training. As shown in Fig. 8 greater initial activation levels were observed in ND compared to depressed groups, and this difference was maintained at frontal and occipitoparietal regions following MT. At baseline D^+^ had great activation than D^−^. Following MT, frontocentral and temporal activation reached ND levels in D^+^, but remained low in D^−^.

The statistical analysis indicates that MT are associated with larger response over the anterior regions for all three groups. This might mean that MT drive activity over the same regions, but drive more and stronger activity for the ND group.

The results were consistent with the recent meta-analysis that found the Mindfulness Based Stress Reduction (MBSR) significantly reduced many parameters, including depression, stress and anxiety scores in those healthy subjects who attended mindfulness programmes compared to waiting list controls^55,56^.

Beyond the ANOVA results, the designed SNN models discovered the differences in the scalp areas involvement between T1 and T2 for three groups. It means that SNN models can learn and identify which areas of the brain contribute to an increase in EEG (at T2) and also how does it happen over time. We could not draw such a conclusion from the ANOVA analysis only. In the current study, the SNN-based methodology is used in integrating the temporal and scalp topographic information, such that a better understanding of the pathways of information processing was obtained, in addition to have discrete measurements of neuronal response.

Although numerous researchers are dedicated to unravelling the functional and structural changes associated with MT, the understanding of the underlying psychological and neural mechanisms is currently limited. The SNN models can be used to obtain new findings, such as tracing a trajectory of neural brain activities, which could not be obtained with the use of traditional statistical methods. The results of previous mindfulness research suggest that meditation improves executive and attentional functioning^3,57,58^. The proposed SNN methodology for modelling mindfulness data confirms these findings, but further extends them to reveal the connectivity intensity across different brain regions affected by mindfulness practice. When compared with traditional machine-learning techniques or deep-learning neural networks, the SNN model has the following advantages: 1) it preserves the spatio and temporal information both together in one model and can be interpreted as this model is spatially structured according to a brain template; 2) It learns spatio-temporal patterns from data through biologically plausible learning rules; 3) It allows for interpretation of the interactions and relationships between the brain data variables. The classification results of EEG patterns learned in an SNN model confirm that the model can precisely discriminate the spatio-temporal patterns of T1 versus T2.

The results of this paper can be further extended to suggest that the effect of MT can be deeply understood through evaluating the both “activation time” and “spiking intensity” in the SNN model across different regions. This will help us to understand how long the brain regions affected by MT can be also last at post-training. If we understand when and where response arise in the brain, we can also understand why and how they affect the other areas of the brain and how they evolve. Analysis of dynamic activated regions involved in the attentional and emotional network can help us to elucidate the process of trajectory of functional changes associated with mindfulness training.

In conclusion, the present study offers an applied SNN-based methodology that enables for a better understanding of how MT could affect individuals’ brain activity with different mental states. The results give strong support for exploring the potential for predicting possible individual responses to mindfulness that precede training. Future application could focus on the clinical setting utilising this model in a practical way to develop optimal and individualised treatment plans that were tailored specifically to the psychological profile and brain architecture of an individual.

## Supplementary information

Supplementary Info

## Data Availability

The data of this study that supports the results are available upon request.
