# Supplementary material for: Spiking Neural Network Modelling Approach Reveals How Mindfulness Training Rewires the Brain
Source: Sci Rep. 2019 Apr 23;9:6367. doi: 10.1038/s41598-019-42863-x (PMC6478904; doi:10.1038/s41598-019-42863-x)
Supplement: Supplementary file 1 — Supplementary Info [file 41598_2019_42863_MOESM1_ESM.pdf]

Spiking Neural Network Modelling Approach Reveals How Mindfulness Training Rewires the Brain

Authors: Zohreh Doborjeh[1]*, Maryam Doborjeh[1], Tamasin Taylor[2] , Nikola Kasabov[1], Grace Y Wang[2] , Richard Siegert[2] , Alex Sumich[3]

Affiliations:

[1]Knowledge Engineering and Discovery Research Institute (KEDRI), Auckland University of Technology, New Zealand

[2]Department of Psychology, Auckland University of Technology, New Zealand

[3]Division of Psychology, Nottingham Trent University, United Kingdom

***Correspondence to:** Zohreh Doborjeh, zgholami@aut.ac.nz

Supplementary Information, Figures, Tables

The paper presents a new biologically-plausible methodology based on deep learning in spiking neural networks (SNN) for knowledge discovery and a better understanding of brain activities before and after Mindfulness training (MT). The proposed SNN is constructed with the use of a brain-template and is used to model Electroencephalography (EEG) data, collected from participants with different levels of depression. We argue that inner patterns, both structural and functional, can be learnt from temporal EEG data when using structural mapping and deep learning in a brain-inspired SNN architecture.

Our findings reveal principally new information when compared with statistical findings reported in previous mindfulness related studies by including both space and time components of data in the model learning. Using 3D visualisation of the SNN model allows for a better interpretation of underpinning brain functions and brain reorganisation under mindfulness training.

Table 1. Information of 18 participants who were affected by different levels of depression from minimal (non-depressed) to severe, according to the Beck Depression Inventory (BDI-II) assessment. Based on BDI-II profile, the severity rating for depression subscale labelled as normal (between 1-10), Mild (between 11-16), clinical depression (between 17-20), Moderate depression (between 21-30), severe depression (between 31-40) and extreme depression (over 40). Non-Depressed group (denoted as ND), depressed group who responded to the training (denoted as D^+) and depressed group who did not respond to the training (denoted as D^-). Descriptive information such as age, gender, mean age, range, mean score and the standard deviation are also reported in the supplementary materials Tables 2 and 3.

Participants	Id	Age	Gender	BDI score at T1	BDI score at T2
**ND Group**	**S##1**	34	F	10	8
	**S##2**	18	F	5	5
	**S##3**	43	F	6	5
	**S##4**	19	F	3	4
	**S##5**	37	F	10	9
	**S##6**	33	F	2	3
	**S##7**	20	F	8	9
**D^+**	**S1**	20	M	20	8
	**S2**	21	F	18	6
	**S3**	30	M	22	3
	**S4**	26	F	18	2
	**S5**	25	M	21	8
	**S6**	24	F	18	6
**D^-**	**S#1**	29	M	22	18
	**S#2**	29	F	36	22
	**S#3**	21	M	20	19
	**S#4**	19	F	22	18
	**S#5**	40	M	22	23

Table 2. Descriptive information of all participants including age, gender, mean age, range, mean score and the standard deviation.

Descriptive Statistics			
		**Male**	**Female**
**N**	**Valid**	6	12
	**Missing**	0	0
**Mean**		27.5	26.9167
**Std. Deviation**		7.342	8.25126
**Variance**		53.9	68.083
**Range**		20	25
**Minimum**		20	18
**Maximum**		40	43

Table 3. Descriptive information of BDI test, including mean and standard deviation.

Descriptive Statistics			
		**BDI score at T1**	**BDI score at T2**
**N**	**Valid**	18	18
	**Missing**	0	0
**Mean score**		15.72	9.78
**Std. Deviation**		8.857	6.924

Table 4. The average of the connection weights between each input neuron and a cluster of neighbouring neurons that are connected to it during the learning process across both left and right sides of five sites: F (Frontal), FC (Frontocentral), T (Temoporal), CP (Cenroparietal) and OP (Occipitoparietal) at T1 (before the treatment) and at T2 (after the treatment) for all the 3 groups: a) non-depressed (ND); (b) responsive-depressed (D^+); and (c) unresponsive-depressed (D^-).The mean and standard deviation (SD) of both hemispheres at T1 and T2 are also reported

Groups	Sites	T1		T2	
		**Left**	**Right**	**Left**	**Right**
**a) ND**	**F**	0.97	1.07	1.24	1.44
	**FC**	0.83	1.02	0.99	0.95
	**T**	0.95	1.14	0.99	1.15
	**CP**	0.68	0.91	0.72	1.12
	**OP**	0.9	0.63	0.97	1.1
**Mean (SD)**		0.86(0.11)	0.95(0.11)	0.98(0.18)	1.15(0.17)
**b) D^+**	**F**	0.88	0.75	1.19	1.03
	**FC**	0.66	0.81	0.1	0.95
	**T**	0.82	0.91	0.99	1.16
	**CP**	0.82	0.86	0.88	1.1
	**OP**	0.63	0.48	0.76	0.53
**Mean (SD)**		0.76(0.11)	0.76(0.17)	0.78(0.41)	0.95(0.24)
**c) D^-**	**F**	0.56	0.52	0.71	0.64
	**FC**	0.49	0.43	0.68	0.67
	**T**	0.66	0.57	0.86	0.72
	**CP**	0.46	0.79	0.41	0.86
	**OP**	0.45	0.22	0.56	0.23
**Mean (SD)**		0.52(0.09)	0.51(0.2)	0.64(0.16)	0.62(0.23)

Table 5. Descriptive information such as mean and standard deviation across all groups (non-depressed (ND); depressed group who responded to the treatment (D^+); and depressed group who did not respond to the treatment (D^-)) for both variables of the research (sites and hemisphere) at T1.

		Descriptive			
**Hem**	**Sites**	**Group**	**Mean**	**SD**	**N**
**Left**	Frontal	ND	0.974	0.180	7
		D+	0.875	0.284	6
		D-	0.555	0.227	5
	Temporal	ND	0.954	0.266	7
		D+	0.825	0.224	6
		D-	0.654	0.313	5
	Frontocentral	ND	0.826	0.343	7
		D+	0.656	0.130	6
		D-	0.488	0.129	5
	Centroparietal	ND	0.682	0.303	7
		D+	0.825	0.180	6
		D-	0.456	0.142	5
	Occipitoparietal	ND	0.900	0.120	7
		D+	0.629	0.125	6
		D-	0.449	0.100	5
**Righ**	Frontal	ND	1.071	0.223	7
		D+	0.747	0.217	6
		D-	0.527	0.071	5
	Temporal	ND	1.136	0.132	7
		D+	0.906	0.174	6
		D-	0.567	0.102	5
	Frontocentral	ND	1.020	0.147	7
		D+	0.812	0.150	6
		D-	0.432	0.166	5
	Centroparietal	ND	0.913	0.226	7
		D+	0.860	0.268	6
		D-	0.787	0.129	5
	Occipitoparietal	ND	0.631	0.169	7
		D+	0.483	0.462	6
		D-	0.219	0.088	5

Table 6. Descriptive information such as mean and standard deviation across all groups groups (non-depressed (ND); depressed group who responded to the treatment (D^+); and depressed group who did not respond to the treatment (D^-)) for both variables of the research (sites and hemisphere) at T2.

**Descriptive**					
**Hem**	**Sites**	**Group**	**Mean**	**SD**	**N**
**Left**	Frontal	ND	1.237	0.169	7
		D+	1.190	0.196	6
		D-	0.701	0.210	5
	Temporal	ND	0.983	0.238	7
		D+	0.987	0.207	6
		D-	0.859	0.543	5
	Frontocentral	ND	0.994	0.146	7
		D+	0.998	0.178	6
		D-	0.676	0.141	5
	Centropareital	ND	0.715	0.142	7
		D+	0.882	0.188	6
		D-	0.409	0.094	5
	Occipitoparietal	ND	0.968	0.126	7
		D+	0.758	0.102	6
		D-	0.564	0.089	5
**Right**	Frontal	ND	1.436	0.090	7
		D+	1.025	0.215	6
		D-	0.639	0.130	5
	Temporal	ND	1.151	0.183	7
		D+	1.160	0.238	6
		D-	0.724	0.131	5
	Frontocentral	ND	0.950	0.122	7
		D+	0.949	0.150	6
		D-	0.666	0.136	5
	Centropareital	ND	1.123	0.181	7
		D+	1.105	0.341	6
		D-	0.857	0.217	5
	Occipitoparietal	ND	1.104	0.266	7
		D+	0.537	0.216	6
		D-	0.233	0.112	5

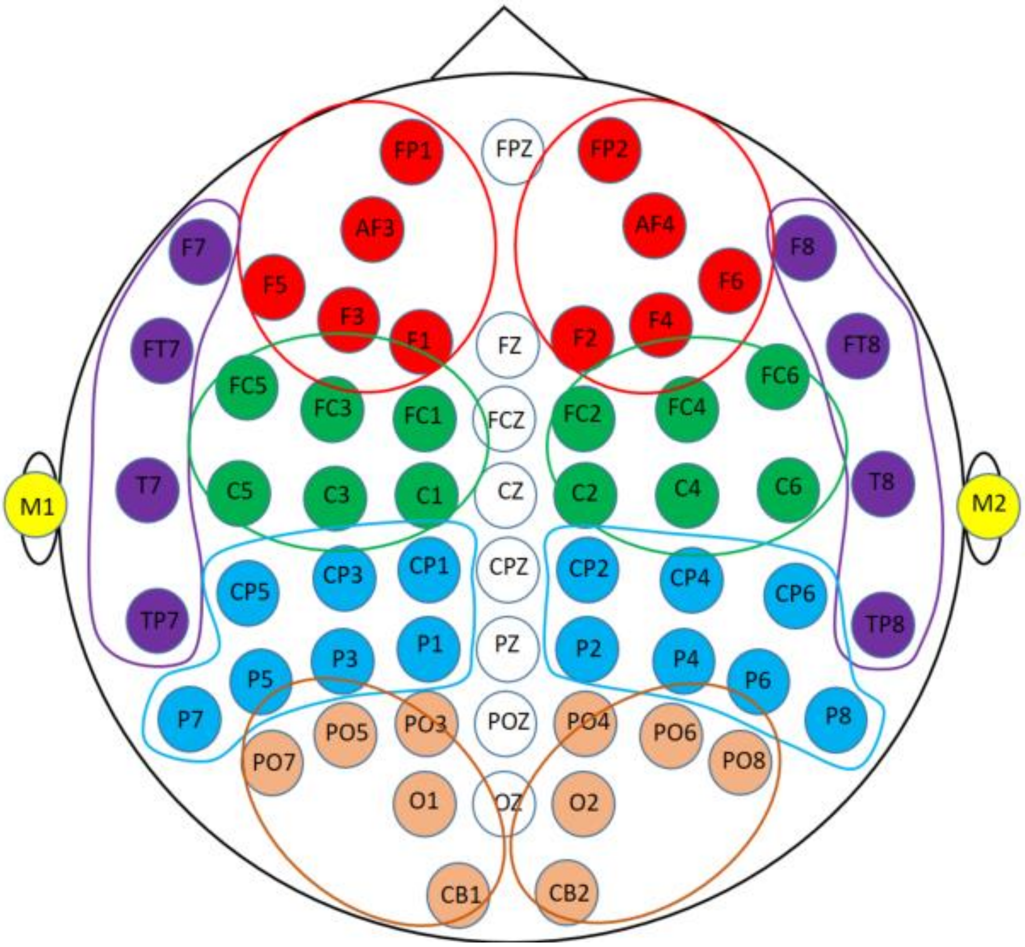

Figure 1. Clustering of EEG channels into five sites for both hemisphere (left and right) with respect to their topological information including: Left Frontal (Fp1, AF3, F5,F3 and F1); Right Frontal (Fp2, AF4, F6, F4 and F2); Left FrontoCentral (FC5, FC3, FC1, C5, C3 and C1); Right FrontoCentral (FC6, FC4, FC2, C6, C4 and C2); Left Temporal (F7, FT7, T7 and TP7); Right Temporal (F8, FT8, T8, TP8); Left CentroParietal (CP5, CP3, CP1, P7, P5, P3, P1); Right CentroParietal (CP6, CP4, CP2, P8, P6, P4 and P2); Left OccipitoParietal (PO7, PO5, PO3,O1 and CB1); and Right OccipitoParietal (PO8, PO6, PO4, O2 and CB2).

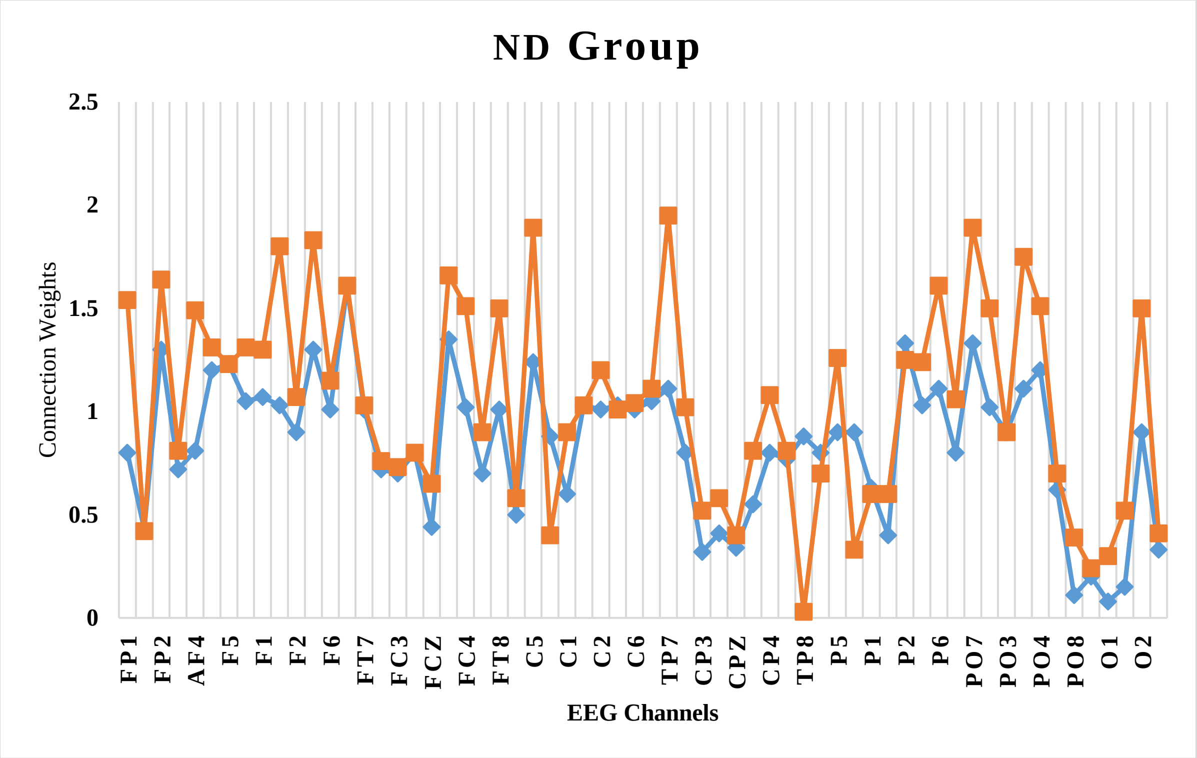

Figure 2. The average of the connection weights created by each of the input neurons (representing an EEG channel) in the SNN models trained on EEG data related to non-depressed participants (ND) showing changes before and after the training. Blue line represents the connectivity values in the SNN model before the training (T1) and orange line represents the ones after the training (T2).

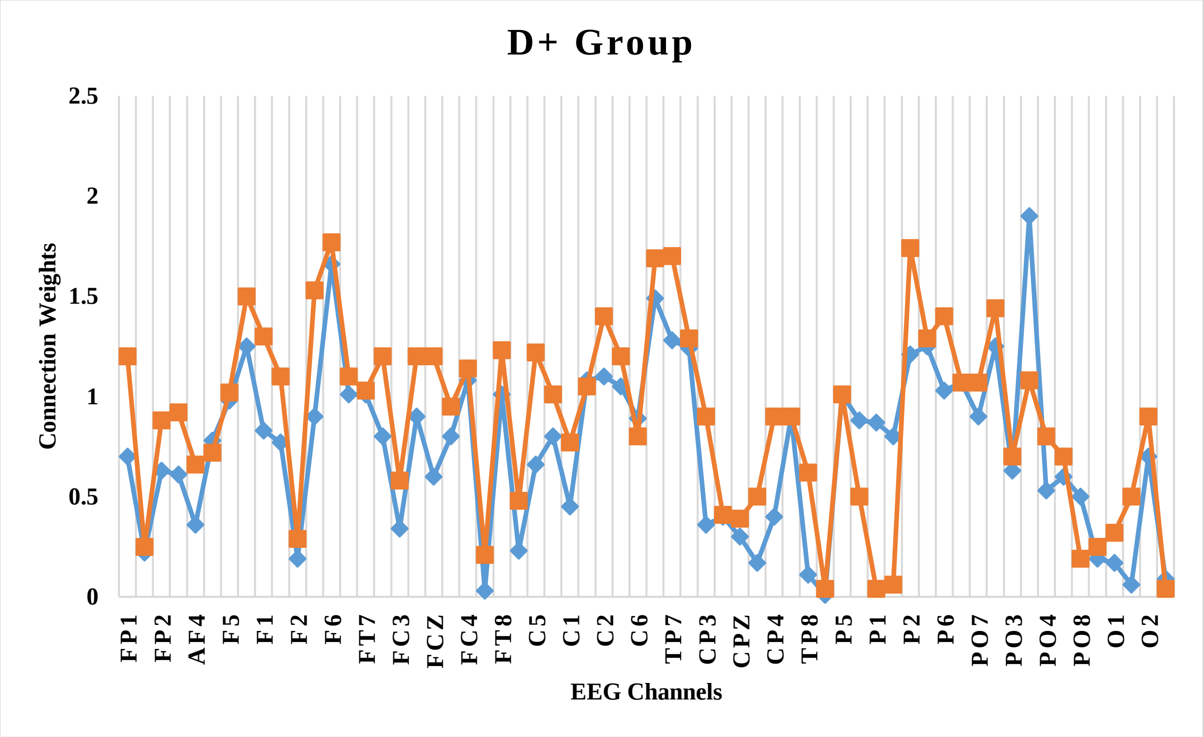

Figure 3. The average of the connection weights created by each of the input neurons (representing an EEG channel) in the SNN models trained on EEG data related to depressed group who responded to the training (D^+) showing changes before and after the training. Blue line represents the connectivity values in the SNN model before the training (T1) and orange line represents the ones after training (T2).

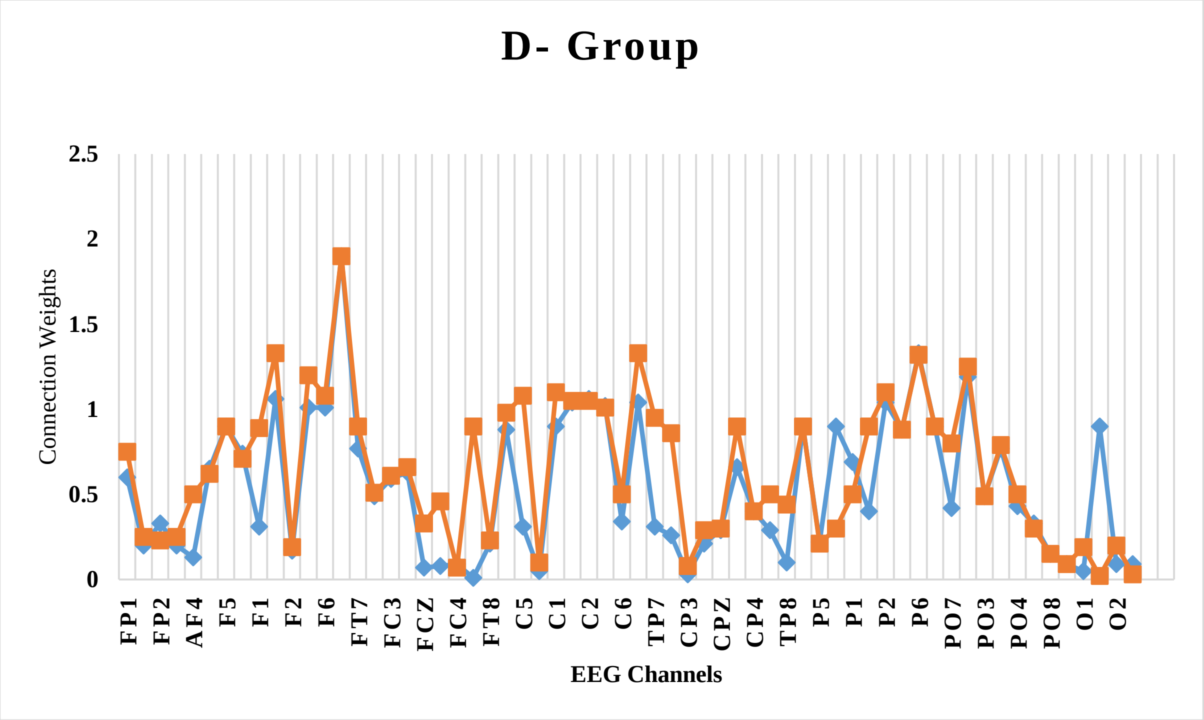

Figure 4. The average of the connection weights created by each of the input neurons (representing an EEG channel) in the SNN models trained on EEG data related to depressed group who did not respond to the training (D^-) showing changes before and after the training. Blue line represents the connectivity values in the SNN model before the training and orange line represents the ones after training.
